# Analysis of hairpin RNA transgene-induced gene silencing in *Fusarium oxysporum*

**DOI:** 10.1186/1758-907X-4-3

**Published:** 2013-07-02

**Authors:** Ulrike Schumann, Neil A Smith, Kemal Kazan, Michael Ayliffe, Ming-Bo Wang

**Affiliations:** 1 Commonwealth Scientific and Industrial Research Organisation Plant Industry, Clunies Ross Street, Canberra ACT 2601, Australia; 2 Commonwealth Scientific and Industrial Research Organisation Plant Industry, Queensland Bioscience Precinct , 306 Carmody Road, St. Lucia QLD 4067, Australia

**Keywords:** RNA silencing, Hairpin RNA, Fungi, Polyadenylation, Convergent promoters

## Abstract

**Background:**

Hairpin RNA (hpRNA) transgenes can be effective at inducing RNA silencing and have been exploited as a powerful tool for gene function analysis in many organisms. However, in fungi, expression of hairpin RNA transcripts can induce post-transcriptional gene silencing, but in some species can also lead to transcriptional gene silencing, suggesting a more complex interplay of the two pathways at least in some fungi. Because many fungal species are important pathogens, RNA silencing is a powerful technique to understand gene function, particularly when gene knockouts are difficult to obtain. We investigated whether the plant pathogenic fungus *Fusarium oxysporum* possesses a functional gene silencing machinery and whether hairpin RNA transcripts can be employed to effectively induce gene silencing.

**Results:**

Here we show that, in the phytopathogenic fungus *F. oxysporum*, hpRNA transgenes targeting either a β-glucuronidase (*Gus*) reporter transgene (*hpGus*) or the endogenous gene *Frp*1 (*hpFrp*) did not induce significant silencing of the target genes. Expression analysis suggested that the hpRNA transgenes are prone to transcriptional inactivation, resulting in low levels of hpRNA and siRNA production. However, the *hpGus* RNA can be efficiently transcribed by promoters acquired either by recombination with a pre-existing, actively transcribed *Gus* transgene or by fortuitous integration near an endogenous gene promoter allowing siRNA production. These siRNAs effectively induced silencing of a target *Gus* transgene, which in turn appeared to also induce secondary siRNA production. Furthermore, our results suggested that hpRNA transcripts without poly(A) tails are efficiently processed into siRNAs to induce gene silencing. A convergent promoter transgene, designed to express poly(A)-minus sense and antisense *G*us RNAs, without an inverted-repeat DNA structure, induced consistent *Gus* silencing in *F. oxysporum*.

**Conclusions:**

These results indicate that *F. oxysporum* possesses functional RNA silencing machineries for siRNA production and target mRNA cleavage, but hpRNA transgenes may induce transcriptional self-silencing due to its inverted-repeat structure. Our results suggest that *F. oxysporum* possesses a similar gene silencing pathway to other fungi like fission yeast, and indicate a need for developing more effective RNA silencing technology for gene function studies in this fungal pathogen.

## Background

RNA silencing is an evolutionary conserved molecular mechanism that functions in genome defense and stability and also plays an important role in developmental regulation [1-3]. This process is characterized by the production of double-stranded RNA (dsRNA) molecules, which are cleaved by a Dicer-like protein (Dcl) into 20 to 25 nucleotide (nt) small RNAs (sRNA) that are subsequently incorporated into an Argonaute protein (Ago) located in the RNA-induced silencing complex (RISC). These sRNAs subsequently enable the RISC to identify complementary mRNA sequences, leading to their targeted degradation by the action of the Ago protein [4,5]. However, not all sRNAs are dependent upon Dicer action and several other classes of sRNAs are produced by less well-characterized pathways that are restricted to certain kingdoms of life in some instances [6-10]. In general, the sRNA pathways in most fungal species have been poorly characterized.

One of the better understood sRNA pathways in eukaryotes is the production of micro RNAs (miRNAs). These sRNA species are derived from endogenous genes and regulate developmental processes via post-transcriptional regulation of gene expression [11]. Only recently miRNA-like genes (milRNA) have been identified in *Neurospora crassa* although their role in this fungal species remains unclear as does their distribution throughout the fungal kingdom [8]. Dicer-independent small interfering RNAs (disiRNAs) and DNA damage-induced Qde2-interacting siRNAs (qiRNAs) have also been identified in *Neurospora crassa*[7,8] but their occurrence in other fungal species is undetermined. In another well-studied fungus, *Schizosaccharomyces pombe* (fission yeast) only a single set of silencing machinery genes exists (for example, a single Dcl and Ago), which appear to function in both transcriptional and post-transcriptional silencing pathways [12-14]. Recent analyses of many fungal genome sequences have revealed that some fungal species, such as the *Candida* species, *Saccharomyces cerevisiae* and *Ustilago maydis*, may have lost the RNA silencing machinery genes entirely [15,16]. In these fungal species alternative RNA-mediated pathways may be present to regulate gene expression. For instance in *Saccharomyces cerevisiae* trans-acting antisense RNAs have been demonstrated to play a role in gene regulation [17,18]. These findings suggest that the fungal RNA silencing pathways may have evolved from a single, common ancestral pathway. The function of RNA silencing in fungal development is poorly understood, and mutation of RNA silencing genes resulted in an obvious phenotype only in few fungal species [19,20]. In contrast, plant and animal RNA silencing mutants, particularly the miRNA pathway mutants, often display severe developmental defects.

The RNA silencing mechanism has been exploited as a tool for gene functional analysis in many eukaryotic organisms, and expression of hairpin-forming transcripts is now reliably used in many animal and plant species to elucidate gene function. Such hairpin RNA (hpRNA) technologies are also the method of choice for a number of fungal species, particularly since gene knock-out mutants can be difficult to obtain in these organisms [21-24]. Whereas in plants and animals expression of hpRNA generally leads to siRNA production, this is not always the case in fungi. Different hpRNA constructs targeting endogenous genes or transgenes have been tested in several fungal species with varying success (reviewed by [22,24]). In addition, hpRNA expression in some fungi such as fission yeast resulted in not only post-transcriptional silencing, but also heterochromatin formation [14,25,26].

In this study, we investigated hpRNA transgene-induced silencing in the fungus *Fusarium oxysporum*. We provide evidence indicating that, although the RNA silencing machinery exists in this fungus, hpRNA transgenes are usually transcriptionally silenced and ineffective at inducing post-transcriptional silencing of target genes. Our study provides new insights into the RNA silencing mechanisms in this fungal pathogen. It also adds to the current understanding of RNA silencing in fungi and supports the notion that RNA silencing processes are more divergent in fungi than in plants or animals, with different fungi possessing alternative mechanisms that may be species specific.

## Results

### Transformation of *Fusarium oxysporum* with a hairpin RNA construct does not result in silencing of a β-glucuronidase reporter gene

**Figure 1:**
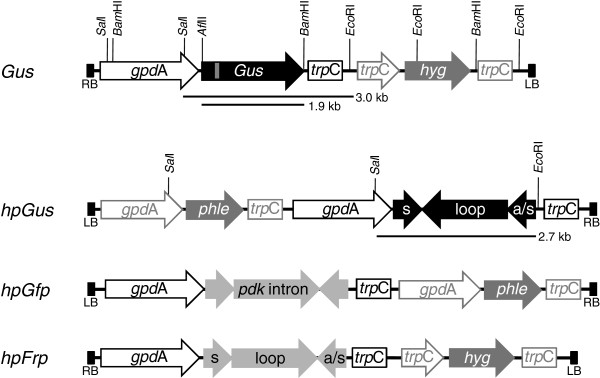
**Schematic diagrams (not to scale) of transgenes introduced into *Fusarium oxysporum*.** All *Gus*-derived sequences are shown in black, except a small region of the *Gus* ORF represented in grey, which is present in the full length *Gus* gene but absent in the *hpGus* constructs. The *gpd*A promoter is shown as an open arrow, while the transcription terminator sequence *trp*C is indicated as an open box. Sequences present in hairpin constructs of the *Gfp* and *F. oxysporum Frp*1 are shown as light grey regions in each construct, respectively. An intron from the pyruvate dehydrogenase kinase gene (*pdk*) is included in the *hpGfp* gene. The hygromycin phosphotransferase gene (*hyg*) and *Streptomyces verticillius* bleomycin gene (*phle*) were used as selectable markers for *F. oxysporum* transformation and are shown as dark grey arrows. The positions and expected fragment sizes of restriction endonuclease recognition sites used for DNA blot analyses are indicated.

**Figure 2:**
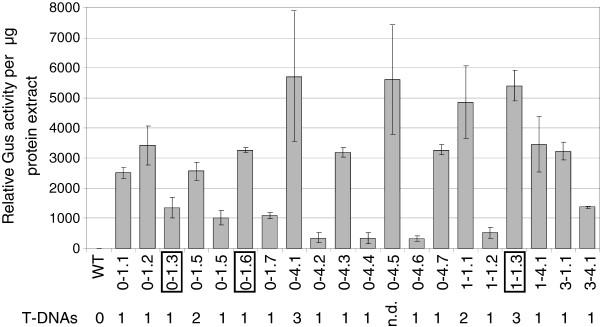
**Relative Gus activity of *Fusarium oxysporum *lines containing the *Gus *gene.** Gus activity was measured by MUG assays of at least three biological replicates of each line. The Y-axis depicts Gus activity per μg of protein extract, while each column on the X-axis represents the activity of a single transgenic line. Standard deviations are indicated on each column. The number of *Gus* T-DNA insertions present in each line is indicated below the X-axis and was determined by DNA blot hybridization using a probe specific for the hygromycin selectable marker gene. Lines 0–1.3, 0–1.6 and 1–1.3 were used for subsequent *hpGus* transformation.

In order to develop a reporter gene system for studying RNA silencing in *Fusarium oxysporum*, the *F. oxysporum* strain 5176 was transformed with a *Gus* construct under the regulatory control of the *gpd*A promoter (Figure 1). Twenty independent lines were isolated and all exhibited varying degrees of Gus activity, as determined by the fluorimetrical assay using 4-methylumbelliferyl-β-D-glucuronide (MUG) (Figure 2). The majority of Gus lines contained a single T-DNA insertion and no correlation between transgene copy number and Gus activity was apparent (Figure 2).

To study hpRNA-induced silencing, wild type (WT) *F. oxysporum*, plus three Gus lines that showed low, intermediate and high Gus activities (lines 0–1.3, 0–1.6 and 1–1.3, respectively), were chosen for super-transformation with a hpRNA *Gus* (*hpGus*) construct (Figure 1). As additional controls, these fungal strains were also transformed with another hpRNA construct (*hpGfp*) (Figure 1), and an empty vector control construct (pKR1). All three constructs contain common *gpd*A promoter and *trp*C terminator sequences. Multiple independent transformants were obtained for all constructs (Table 1) and mycelial fractions were analyzed for Gus activity. Super-transformants of Gus lines 0–1.3 and 0–1.6 showed a mixture of Gus positive and negative colonies regardless of the construct used for transformation. In contrast all, except one, super-transformants of line 1–1.3 maintained Gus activity (Table 1).

**Table 1:** **Summary of the β-glucuronidase** (**Gus) activity of all obtained transformants carrying the *hpGus*, *hpGfp *or empty vector control (pKR1)**

***Fusarium*line**	**Control (pKR1)**	***hpGfp***	***hpGus***
WT^a^	11 white	14 white	5 white
0-1.3^a^	4 white 1 blue	3 white 9 blue	1 white 5 blue
0-1.6^a^	11 white 1 blue	12 white 22 blue	8 white 27 blue
1-1.3^a^	0 white 27 blue	0 white 41 blue	1 white 26 blue

^a^Mycelia fractions were incubated with X-glucuronide solution at 37°C overnight and the number of white and blue colonies counted. It is worth noting that only white or dark blue, but not light blue staining was observed.

The absence of Gus activity in the Gus-negative lines could be due to either silencing of the *Gus* gene by the *hpGus* construct, or loss of the *Gus* target gene by homologous recombination with the super-transformed constructs that share the common promoter and terminator sequences. PCR analysis was undertaken on all 0–1.3 and 0–1.6 *hpGus* transformants (additionally, on all 0–1.3 *hpGfp* and control transformants, as well as on four 0–1.6 control and seven 0–1.6 *hpGfp* transformants) to determine if the colonies that do not display Gus activity had also lost the *Gus* transgene. A region unique to the *Gus* transgene was used for PCR analysis and amplification products were only obtained from transformants that maintained Gus activity (data not shown). This result indicated that the absence of *Gus* expression was due to loss of the *Gus* target gene but not due to *hpGus*-induced silencing. The occurrence of Gus-negative super-transformants in the 0–1.3 and 0–1.6 but not the 1–1.3 background is likely because lines 0–1.3 and 0–1.6 only carry a single T-DNA insertion that can be deleted by a single recombination event, whereas line 1–1.3 contains three separate T-DNA insertions, which are unlikely to be all lost by recombination events.

### Intact hairpin RNA transgenes do not produce detectable levels of small interfering RNAs in *Fusarium oxysporum*

**Figure 3:**
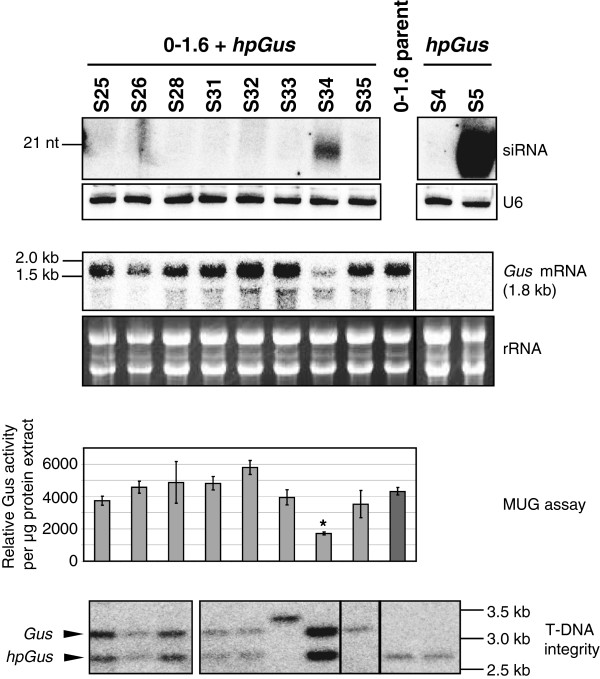
**Analysis of β-glucuronidase hairpin RNA (*hpGus*) transformants of β-glucuronidase reporter transgene (*Gus*) line 0–1.6 and wild type (WT) *Fusarium oxysporum*.** Identification of small RNAs produced in *hpGus* transgenics by RNA blot analysis (top panel). Total RNA (15 μg) was separated on a 17% polyacrylamide gel and probed for *Gus* siRNAs. Numbers above each lane designate an independent *hpGus* transformant in either 0–1.6 parent (left) or WT (right). As a loading control the same membrane was hybridized with a probe specific for the U6 transcripts and is shown below. RNA blot detection of *Gus* transcripts in the analyzed transgenic lines (second panel). Total RNA (10 μg) was hybridized with a probe specific for the region unique to *Gus*. The position of this unique region in the *Gus* gene is indicated in Figure 1, for further details see Methods. The ethidium bromide stained ribosomal RNA bands are shown as loading control. Gus activity of 0–1.6 *hpGus* transformants (third panel) was determined by MUG assay. Shown is the relative Gus activity per μg of protein extract for each transgenic line. Error bars indicate standard deviation of at least two independent biological replicates. MUG assay of line S34, indicated with an asterisk, shows significantly reduced Gus activity (t-test: p = 0.004). DNA blot analysis of *hpGus* transformants of line 0–1.6 to determine integrity of the *Gus* and *hpGus* transgene loci (bottom panel). Genomic DNAs were digested with *Eco*RI and *Sal*I and hybridized with a full length *Gus* probe. Intact *hpGus* and *Gus* transgenes produce conserved 2.7 kb and 3.2 kb restriction fragments, respectively.

Several *hpGus* and *hpGfp* transformants of WT and *Gus* lines 0–1.3 and 0–1.6 were analyzed for the presence of siRNAs expected to be derived from processing of the hpRNA transcripts from these transgenes. No line producing *Gfp*-specific siRNAs was identified out of the nine *hpGfp* transgenics examined (Table 2). Among the 28 *hpGus* lines analyzed, the majority (25) did not show siRNA accumulation (Table 2, Figure 3 and [see Additional file 1: Figure S1]). Only three *hpGus* lines produced detectable levels of *Gus*-specific siRNAs; S5 (in the WT *F. oxysporum* background), S14, and S34 (both in the 0–1.6 background), hereafter referred to as S5, S14 and S34, respectively. MUG assays indicated that a significant reduction in Gus activity was apparent in S34 extracts along with reduced levels of *Gus* mRNA (Figure 3).

**Table 2:** **Summary of all transformants analyzed regarding the production of siRNAs and rearrangement of the β-glucuronidase** overexpression (***Gus***) **and hairpin RNA** (***hpGus***) ** transgene loci**

	**Strains analyzed**	**Strains with siRNAs**	**Strains with*Gus*locus rearrangement**
WT *hpGus*	5	1 (S5)^a^	n.a.
0-1.3 *hpGus*	5	0	n.d.
0-1.6 *hpGus*	18	2 (S14, S34)	3 (S13, S14, S34)
WT *hpGfp*	3	0	n.d.
0-1.3 *hpGfp*	3	0	n.d.
0-1.6 *hpGfp*	3	0	n.d.
WT *hpFrp*	9	8	n.a.
0-1.6 *conP-Gus*	9	0	0

^a^Lines that showed production of *Gus*-specific siRNAs or rearrangement following super-transformation are indicated in parentheses. n.a. not applicable, n.d. not determined, WT wild type.

To further characterize the *hpGus* lines, genomic DNA was digested with *Eco*RI and *Sal*I and hybridized with a *Gus* probe, to detect two conserved fragments corresponding to the *Gus* and *hpGus* transgenes, respectively, if these genes were intact (Figure 1). All lines that did not show siRNA accumulation were found to have intact *hpGus* transgenes (Figure 3 and [see Additional file 1: Figure S1]). However, for S34, which produced significant amounts of siRNAs, both the *Gus* and *hpGus*-specific restriction fragments were absent, and instead, a large hybridizing band was observed (Figure 3), indicating that recombination had occurred between the pre-existing *Gus* transgene locus and the incoming *hpGus* transgene. Similarly, recombination also appeared to have occurred in line S14 [see Additional file 1: Figure S1]. As shown below, line S5, which was the only WT *hpGus* transformant generating *Gus*-specific siRNAs, contains a distinct T-DNA insertion pattern that allowed transcription of the *hpGus* sequence by an endogenous element. Thus, our result showed that intact hpRNA transgenes do not produce detectable amounts of siRNAs, and only transgenic lines with particular T-DNA structures or insertion patterns give rise to siRNAs.

### Small interfering RNA accumulation correlates with the presence of double-stranded RNA precursor

**Figure 4:**
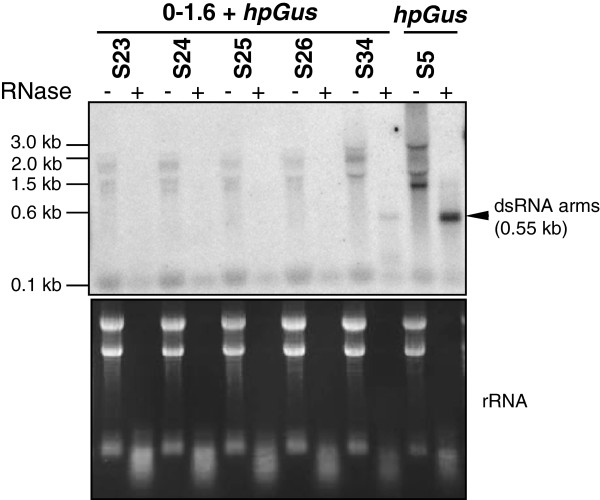
**Double-stranded *Gus *RNA is detected in lines that produce small interfering RNAs (siRNAs).** Total RNAs from *hpGus* transgenic lines was treated either with (+) or without (−) RNase One prior to RNA blot analysis. Hybridization with a full length *Gus* probe detected a 0.55 kb fragment only in strains which produce siRNAs (that is, S34 and S5). The 0.55 kb RNA fragment is derived from annealing of the complementary arms of *hpGus* precursor to form a 0.55 kb dsRNA fragment that is resistant to RNase One digestion. Shown below is the ethidium bromide-stained RNA gel used for hybridization, demonstrating equivalent loading and extensive RNA cleavage following RNase One digestion.

It was previously shown that unprocessed hpRNA or dsRNA can be detected using northern blot hybridization in hpRNA lines of plants accumulating siRNAs [27,28]. We therefore used northern blot hybridization to examine if siRNA accumulation in the *hpGus F. oxysporum* lines was correlated with the expression of dsRNA. RNA samples were treated with RNase One and hybridized for the presence of a 550 nt antisense *Gus* fragment, equivalent to the size of the *hpGus* dsRNA arm. The predicted nuclease-resistant RNA fragment was only detected in RNA from lines that produced siRNAs (that is, S34 and S5), and not in lines that produced no detectable siRNAs (Figure 4). However, hybridizing signals were detected in untreated RNA samples of all siRNA-negative lines tested (S23 to S26) (Figure 4). The pattern of these hybridizing bands was equivalent to the pattern observed in plants expressing the same *hpGus* transcript [27], indicating that *hpGus* is expressed in these fungal lines. However, the level of the hybridizing signals were low compared to the siRNA-generating lines (S34 and S5), suggesting that the *hpGus* transgene is poorly transcribed in the siRNA-negative lines. The *gpd*A promoter driving the *hpGus* was PCR amplified and sequenced in these siRNA-negative lines and found to be unaltered (data not shown), indicating that the low level of *hpGus* transcription was not caused by sequence changes in the promoter. Additionally, DNA blot analysis confirmed the presence and integrity of both *hpGus* and *Gus* transgenes in the transformants analyzed (Figure 3 and [see Additional file 1: Figure S1]). These data suggest that the *hpGus* transgene is usually subject to transcriptional inactivation in *F. oxysporum*, generating insignificant amounts of hpRNA precursor for siRNA production, and that the dsRNA precursor detected in lines S5, S14 and S34 are derived from transgenes in a specific genomic context(s) that enables active hpRNA or dsRNA transcription. These data also indicate that *F. oxysporum* possesses the necessary machineries for processing dsRNA or hpRNA into siRNAs.

### A hairpin RNA transgene targeting an endogenous gene does not induce effective silencing in *Fusarium oxysporum*

**Figure 5:**
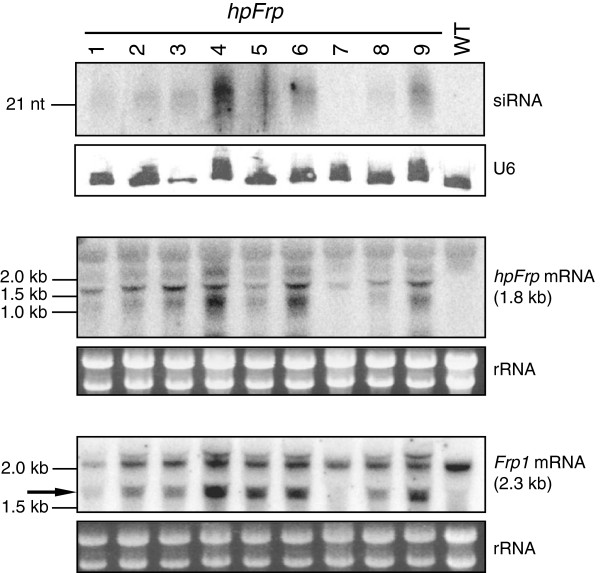
**Endogenous genes can be targeted by hpRNA-derived small interfering RNAs (siRNAs).** Wild type (WT) *Fusarium oxysporum* was transformed with a hpRNA construct directed against the endogenous *Frp1* gene. Total RNA (15 μg) from nine independent transgenic lines was separated on a 17% polyacrylamide gel and hybridized with a probe specific for *Frp*1. The *hpFrp*-derived siRNAs were detected in most lines although the levels are very low (upper panel). The U6 transcripts are shown as loading control. Total RNA (10 μg) was separated on an agarose gel and hybridized with an *Frp*1 sense probe to detect antisense sequences of the *hpFrp* transgene (middle panel). To detect *Frp*1 mRNA levels, total RNA (10 μg) was hybridized with a probe specific for the 3′ region of the endogenous *Frp*1 gene, which is not present in the *hpFrp* gene, detecting 2.3 kb *Frp*1 mRNA, but not *hpFrp* transcripts (lower panel). Ethidium bromide-stained ribosomal RNA is shown as loading control. The additional transcripts detected are likely to be either *Frp*1 mRNA cleavage products (below the endogenous transcript band) or size mobility shifted endogenous *Frp*1 likely due to binding of small RNAs (above the endogenous transcript band), as both are not present in the WT sample.

In addition to the *Gus* reporter gene, we also tested the efficacy of hpRNA-induced silencing on an endogenous gene, *Frp*1. *Frp*1 was chosen as a target because *F. oxysporum* with a loss-of-function mutation of this gene has been shown to be nonpathogenic on tomato [29,30]. Wild type *F. oxysporum* was transformed with an *hpFrp* transgene (Figure 1), and nine independent transgenic lines were chosen for subsequent analyses. Both precursor *hpFrp* transcripts as well as *Frp*-specific siRNAs were detected in eight of these lines, and levels of the siRNAs and precursor hpRNA were correlated (Figure 5; top and middle panels). Again this indicates that *F. oxysporum* possesses the functional RNA silencing machineries required for siRNA biogenesis. However, the abundance of siRNA again appeared low. Furthermore, a strong reduction in endogenous *Frp*1 mRNA levels was not observed in any of the eight lines, presumably as a consequence of the low siRNA levels (Figure 5; bottom panel). Target mRNA analysis using northern blot hybridization detected smaller sized (approximately 1.7 kb) hybridizing bands (indicated by an arrow), which were absent in the WT control and in *hpFrp* line 7 that had no detectable levels of siRNAs (Figure 5; bottom panel). These bands likely represent cleavage products of the *Frp1* mRNA, suggesting that siRNA-mediated cleavage had occurred, although further experiments are needed to characterize the fragments. Taken together, the data on the *hpFrp* transgenic lines further suggest that hpRNA transgenes are not highly expressed in *F. oxysporum* and therefore do not generate sufficient levels of siRNAs required for effective silencing of target genes.

### Small interfering RNAs can mediate target messenger RNA downregulation and induce secondary small interfering RNA production in *Fusarium oxysporum*

S34 was the only transgenic line identified in which significant downregulation of the target *Gus* gene was associated with the accumulation of *Gus*-specific siRNAs (Figure 3). However, DNA blot analysis indicated the absence of both the *Gus* and *hpGus*-specific restriction fragments (Figure 3), suggesting that the target *Gus* gene had undergone rearrangement following super-transformation. Therefore, the reduction in Gus activity in line S34 may be due, in part, to changes in gene expression following this transgene rearrangement rather than a direct result of siRNA-mediated mRNA cleavage.

**Figure 6:**
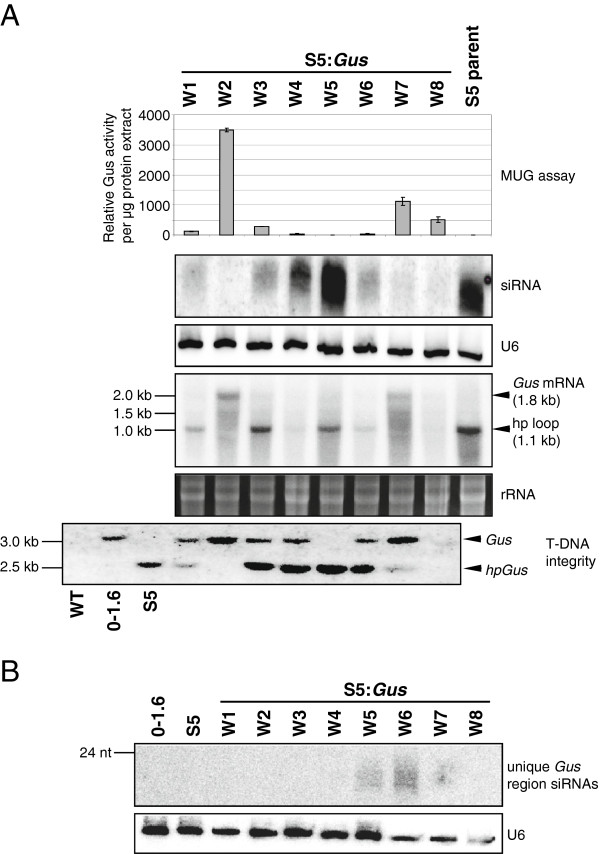
**β-glucuronidase hairpin RNA (*hpGus*)-derived small interfering RNAs (siRNAs) can mediate target transcript downregulation. (A)** Strain S5, which contains the *hpGus* transgene and produces siRNAs (see Figure 3), was super-transformed with the *Gus* transgene to obtain S5:*Gus* lines W1 to W8. Relative Gus activity was determined by MUG assay (top panel). The mean of at least two independent biological replicates is shown with error bars representing the standard deviation. The second panel shows RNA blot analysis of 15 μg of total RNA hybridized with a full length *Gus* probe to detect small RNAs. The U6 transcripts are shown as loading control. Expression levels of the *Gus* and *hpGus* transgenes are shown in the fourth panel. Total RNA (10 μg) was hybridized with a full length *Gus* probe, detecting the1.8 kb transcript derived from the *Gus* transgene and the 1.1kb fragments corresponding to the single stranded loop region of the *hpGus* transcript. Ethidium bromide-stained ribosomal RNA bands are shown as loading control. DNA blot analysis was performed to determine transgene integrity (bottom panel). Genomic DNA was restricted with *Eco*RI and *Sal*I, and hybridized with a full length *Gus* probe. Restriction fragments corresponding to the *hpGus* (2.7 kb) and *Gus* transgenes (3.2 kb) are present in most lines, indicating that both transgenes remain intact. **(B)** Secondary siRNAs are produced in some of these lines. Total RNA (15 μg) from S5:*Gus* lines W1 to W8 were resolved on 17% polyacrylamide and hybridized with a probe specific for the unique region that is present only in the *Gus* but not the *hpGus* transcript (see Figure 1 and Methods for details). Low levels of *Gus*-specific siRNAs were identified in lines W5 to W7. The U6 transcript is shown as loading control.

We therefore investigated if siRNAs are capable of inducing effective silencing in *F. oxysporum* by super-transforming line S5, which showed high levels of *Gus*-specific siRNAs, with the *Gus* construct and subsequently measuring the *Gus* expression levels in the resulting super-transformants. As shown in Figure 6A, with the exception of line S5:*Gus* W2, all of the eight S5:*Gus* super-transformants showed greatly reduced *Gus* expression in comparison to the *Gus* lines shown in Figure 2. Furthermore, the level of *Gus* mRNA was in general inversely correlated with the level of *Gus*-specific siRNAs (Figure 6A).

To exclude the possibility that the low *Gus* mRNA levels were due to transgene rearrangement, DNA blot analysis was performed. Five out of the eight S5:*Gus* super-transformants contained the predicted *Gus* and *hpGus*-specific restriction fragments (Figure 6A). These five super-transformants showed low levels of Gus activity as well as low levels of *Gus* mRNA along with the presence of siRNAs, indicating that the reduced *Gus* expression is due to siRNA-mediated mRNA cleavage. To demonstrate that RNA silencing was occurring in these lines, *Gus* mRNA cleavage products were cloned from RNA of line S5:*Gus* W4 using 5′ RACE (see Methods). Several individual fragments were sequenced and found to represent four distinct cleavage sites within the *Gus* mRNA, indicating that siRNA-mediated target mRNA cleavage had occurred (see below and Table 3).

**Table 3:** **Cleavage products obtained by 5′ RACE of RNA samples from line S5: *Gus *W4**

**Cleavage site in*Gus*mRNA (nt)**^**c**^	**Number of fragments sequenced**^**d**^
80 | 81^b^	3
428 | 429^b^	5
548 | 549^b^	7
587 | 588^a^	6
623 | 624^a^	3
778 | 779^a^	1

*Gus,* β-glucuronidase, *nt* nucleotides.

^a^Nucleotides 556–792 correspond to the unique region of the *Gus* gene. ^b^The first 555 nucleotides of the *Gus* gene were used to construct the double stranded arm region of the *hpGus* gene. ^c^Shown are the nucleotide (nt) numbers of the *Gus* gene between which cleavage had occurred and ^d^how often this fragment was recovered.

It is noteworthy that in all five lines that contained both the *Gus* and *hpGus* transgenes, the levels of siRNAs were lower than in the initial parental line S5. This reduction in siRNA level implies that the presence of target mRNA may destabilize complementary small RNAs in *F. oxysporum*. This possibility is consistent with the observations that expression of miRNA target mimic transcripts reduces the level of the respective miRNA in plants [31] and that miRNA decay rates are dramatically enhanced by the presence of highly expressed target genes in human cells [32].

*F. oxysporum* S5:*Gus* transformants were also used to investigate whether an amplification mechanism, through which secondary siRNAs are generated, exists in this fungal species. A probe that is specific for the unique region of the *Gus* transgene, and therefore does not recognize the *hpGus* sequence (see Methods), was hybridized to RNA from the eight S5:*Gus* lines. Low levels of small RNAs derived from this unique region of the *Gus* transgene were detected in the S5:*Gus* lines W5 and W6 (Figure 6B). These small RNA species were derived from sequences outside of the *hpGus* transgene and are therefore likely to be generated by an amplification mechanism. The lack of such siRNAs in the other S5:*Gus* lines could be explained by different transgene insertions having different susceptibility to silencing amplification as observed for transgenes in plants (for example, [33]). However, this result does not exclude the possibility that the small RNAs detected in lines W5 and W6 were generated due to specific integration patterns of the newly introduced *Gus* transgene alone, but independently of the pre-existing *hpGus* transgene.

### Hairpin RNA is transcribed from recombined promoters

As described above, lines S14 and S34 each contained a single, aberrant restriction fragment in DNA blot experiments, when hybridized with a *Gus*-specific probe, whereas the conserved *Gus* and *hpGus*-specific fragments were absent (Figure 3 and [see Additional file 1: Figure S1]). In each line this unique restriction pattern is likely to be a consequence of *hpGus* transgene integration within the *Gus* transgene, such that Gus activity was lost in S14, but retained in S34, and both strains were capable of producing siRNAs (Figure 3 and [see Additional file 1: Figure S1]). To determine the nature of this insertion event in line S34, a lambda phage library was created and a phage colony which contained the entire *Gus* locus isolated and sequenced in its entirety.

**Figure 7:**
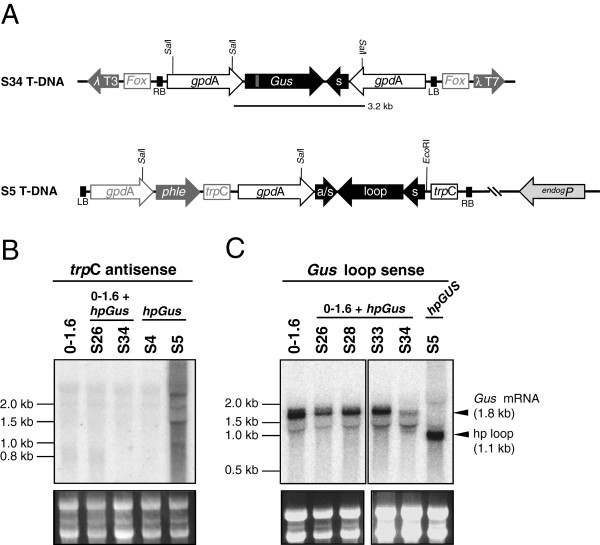
**Analysis of the transgene re-arrangments in *Fusarium oxysporum *lines S34 and S5. (A)** Schematic diagrams (not to scale) of the transgenes in *F oxysporum* lines S34 and S5. The structure of the S34 locus was determined by sequencing of a *lambda phage* clone containing this entire region and was derived likely by recombination between the pre-existing *Gus* transgene and an incoming *hpGus* transgene, such that the full length *Gus* ORF is followed by the 550 nt antisense *Gus* arm and the *gpd*A promoter, both derived from the *hpGus* transgene. The resulting hairpin-like *Gus* sequence is flanked by convergent *gpd*A promoters. Details of both transgenes prior to the recombination event are shown in Figure 1. *Fox*, *F. oxysporum* genomic sequences; λT3 and λT7, *lambda phage* T3 and T7 RNA polymerase binding sites. **(B)** The *hpGus* transcripts in strain S5 were likely derived from an endogenous promoter 3′ of the T-DNA insertion site. Total RNA (10 μg) from the *Gus* 0–1.6 parent (left lane), 0–1.6 *hpGus* lines (middle two lanes) and WT *hpGus* lines (right two lanes) was hybridized with a probe detecting antisense *trpC* terminator sequences. *TrpC* antisense sequences were only present in line S5, suggesting that these transcripts are produced by an endogenous promoter located downstream of the *hpGus* integration site. **(C)** Hybridization of total RNA (10 μg) with an antisense *Gus* probe specific for the loop region of the *hpGus* transgene, detecting transcripts that contain sense *Gus* sequences. Transcripts derived from the resident *Gus* transgene (1.8 kb) were detected in all samples except S5, which does not carry the *Gus* transgene. The 1.1 kb *Gus* sequence detected only in RNA of S5, corresponds to the hairpin loop region, likely produced by dicer processing of a correctly folded hairpin transcript.

Sequencing indicated that line S34 carried an inverted repeat of the *Gus* sequence created by integration of the *gpd*A promoter, together with the upstream half of the dsRNA arm of the *hpGus* construct, in antisense orientation downstream of the full-length *Gus* ORF (Figure 7A). This rearrangement is consistent with a single 3.2 kb *Sal*I fragment being present in genomic DNA of this line upon DNA blot hybridization with a *Gus* specific probe (Figure 3). Given the rearrangements apparent at this locus and the significant level of Gus activity, it is likely that the RNA is transcribed by the *gpdA* promoter of the resident *Gus* target gene, and that this RNA encodes both, a functional Gus protein and a hpRNA template for siRNA production. Presumably this transcription is inefficient, explaining the comparatively low siRNA and *Gus* transcript levels in this line (Figure 3).

Next we investigated why hpRNA was efficiently transcribed in line S5. As the *gpd*A promoter of the *hpGus* transgene generally did not produce high levels of *hpGus* RNA and *Gus*-specific siRNAs (Figure 3 and [see Additional file 1: Figure S1]), it was possible that siRNAs present in line S5 were derived from an endogenous promoter downstream of the T-DNA insertion site (Figure 7A). RNA blot analysis using a probe specific for *hpGus* precursor transcripts detected a smear of hybridizing signals in S5 RNA (Figure 4), indicating the expression of *hpGus* RNA of varying size. When RNAs were hybridized with a probe specific for antisense *trp*C terminator sequences (Figure 1, Figure 7A), multiple transcripts were detected in line S5, which were absent in other *hpGus* transformants (Figure 7B). This indicated that transcription occurred in the opposite orientation to the *gpdA* promoter of the *hpGus* transgene, presumably by an endogenous promoter adjacent to the T-DNA insertion site. Several attempts of tail-PCR were made to clone the flanking endogenous sequence but were unsuccessful. The presence of multiple hybridizing bands is likely due to the absence of a transcription termination signal that can stop transcription from the endogenous promoter. Similarly, when RNAs were hybridized with a probe that would only detect sense *hpGus* loop sequences (hence indicating transcription from the opposite direction), a one-kilobase fragment, consistent with the size of a processed sense loop transcript, was present in line S5 RNA, but not in RNAs of other *hpGus* transformants (Figure 7C). Such full-length processed loop fragments are typical of hpRNA expressed in plants [28]. Thus, in both lines S34 and S5, hpRNA appears to be transcribed by an endogenous promoter gained through specific transgene integration events from a resident transgene or endogenous gene, but not by the transgenic promoter of the original *hpGus* transgene cassette.

### Consistent induction of RNA silencing by a convergent promoter construct

**Figure 8:**
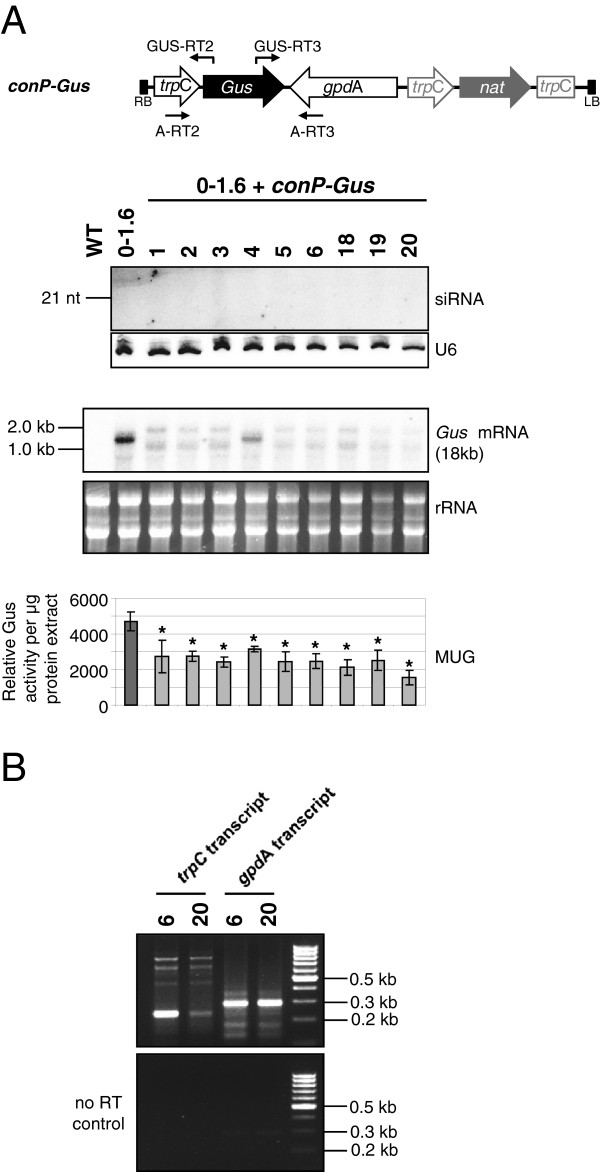
**Analysis of β-glucuronidase (*Gus*) 0**–**1.6 transgenics carrying the *conP-Gus *constructs. (A)** Schematic diagram (not to scale) showing details of the T-DNA region of the *conP-Gus* construct. The *Gus* sequence consists of the 3′ 1.1 kb of the *Gus* ORF and is shown in black. The convergent promoters driving transcription are shown as open arrows. The *Streptomyces noursei* nouseothricin gene was used as selectable marker (clonNAT, Werner BioAgents, Germany) and is shown in grey. Total RNA (15 μg) was separated on 17% polyacrylamide gels and probed for *Gus*-derived small interfering RNAs (siRNAs) (upper panel). No small RNA species were detected in any of these lines. U6 transcripts are shown as loading control. To determine *Gus* transcript levels, total RNA (10μg) was separated by agarose gel electrophoresis and hybridized with a probe specific for the region unique to the *Gus* transgene, not present in the *conP-Gus* gene (middle panel). Most lines show reduced *Gus* mRNA levels. Detected fragments are likely either cleavage products (below the *Gus* fragment) or size shifted due to siRNA binding (above the *Gus* fragment). Ribosomal RNA bands are shown as loading control. All transgenic lines were analyzed for Gus activity, which was carried out by MUG assay in at least two independent biological replicates (bottom panel; error bars show standard deviation). All *conP-Gus* transformants showed significantly reduced Gus activity (*t-test: *P* < 0.003). **(B)***Gus* transcription occurred from both transgenic promoters. Total RNA (500 ng) was reverse transcribed using *Gus*-specific primers *Gus*-RT2 or *Gus*-RT3 (see schematic). Fragments were amplified from cDNA or no RT control RNA using primers *Gus*-RT2 and A-RT2 (*trp*C transcript), or *Gus*-RT3 and A-RT3 (*gpd*A transcript). Products were separated on a 2% agarose gel. Fragments of the correct size were obtained for both promoters, indicating that dsRNA could be produced in these lines.

Our analyses of the *hpGus* lines raised two possibilities. First, hpRNA transgenes in the *F. oxysporum* genome are highly susceptible to transcriptional inactivation, possibly due to the inverted repeat DNA structure, resulting in lack of siRNA production. Second, based on the analyses of lines S34 and S5, dsRNA transcribed from a terminatorless transgene, which would lack polyadenylation, may be more efficiently processed into siRNAs. To test these possibilities, a construct was generated (*conP-Gus*; Figure 8A), which contained two convergent promoters that bi-directionally transcribe a 1.1 kb sequence of the *Gus* ORF to generate dsRNA. This construct contained no terminator sequences and therefore both sense and antisense *Gus* transcripts, were expected to lack poly(A) tails. The construct was transformed into *F. oxysporum* line 0–1.6, which contains an actively expressed *Gus* gene.

All nineteen independent *F. oxysporum* lines carrying the *conP-Gus* construct analyzed showed a significant reduction in Gus activity as determined by MUG assays, in addition to exhibiting greatly reduced *Gus* mRNA levels (Figure 8A). No evidence of transgene rearrangement was observed by DNA blot analysis in any of these lines (data not shown). The observed reduction in Gus activity and *Gus* mRNA levels in *conP-Gus* lines is therefore likely to be a consequence of dsRNA-induced RNA silencing. Consistent with this, the *Gus* sequence of the *conP-Gus* construct was found to be transcribed in both sense and antisense orientation by the convergent promoters (Figure 8B), indicating the likelihood of *Gus* dsRNA formation. The uniform expression of the sense and antisense RNAs across the two independent lines analyzed, suggests that this transgene is not as prone to transcriptional inactivation as the *hpGus* transgene, possibly due to a lack of the inverted repeat DNA structure. However, *Gus*-specific siRNAs could not be clearly detected in any of the *conP-Gus* transformants (Figure 8A), even after small RNA enrichment [see Additional file 1: Figure S3], presumably because siRNA levels were extremely low. This implies that *in vivo* formation of dsRNA through annealing of two separate RNA transcripts is less efficient than through folding of two complementary sequences within the same hpRNA transcript. This is consistent with the observation in plants where co-expression of sense and antisense RNAs from two separate transcription units is generally less effective in inducing target gene silencing [34].

As a comparison to *conP-Gus*, we also transformed line 0–1.6 with a construct that would allow transcription of a sense *Gus* sequence with a poly-A tail plus convergent transcription of an antisense *Gus* sequence without a terminator sequence (*conP-Gus-ter*; [see Additional file 1: Figure S2A]). However, we could not detect transcription past the *trp*C terminator sequence from the *gpd*A promoter [see Additional file 1: Figure S2B] possibly due to a bi-directional transcription termination property of the *trp*C terminator. Also, no significant reduction in *Gus* mRNA levels or Gus activity was observed in these transgenic lines [see Additional file 1: Figure S2C]. This result suggests that the transcription of both sense and antisense *Gus* RNA is required for the observed *Gus* silencing with the *conP-Gus* construct. The lack of antisense *Gus* transcription by *conP-Gus-ter* construct prevented us from examining if the addition of ploy(A) might inhibit the silencing-inducing effect of the converging construct.

## Discussion

The results presented in this paper demonstrate that unlike Ascomycete fungi (reviewed by [22,24]) hpRNA transgenes do not reliably lead to the production of siRNAs in *F. oxysporum*. Neither *hpGus* nor *hpGFP* transgenes were found to produce siRNAs in this species. Transformation with an hpRNA construct targeting the endogenous *Frp1* gene did lead to siRNA production in the majority of analyzed transformants; however, the abundance of siRNAs was low and no strong silencing of the *Frp1* gene was observed. In *Neurospora crassa*, the arm length of the hpRNA constructs was critical for efficient silencing [35]; however this was not a factor affecting the *hpGus* and *hpGfp* constructs used in this study, which were well within these design parameters. The inclusion of a spliceable intron into the hairpin loop region, which has been shown to promote efficient siRNA processing in plants [36], also did not induce siRNA production in *F. oxysporum* (Table 2).

However, our results indicate that *F. oxysporum* does possess functional RNA silencing machineries which process hairpin precursor transcripts into siRNAs that target homologous mRNA for cleavage. Introduction of the *hpFrp* transgene led to production of siRNAs in the majority of lines, resulting in cleavage of the target *Frp1* mRNA, although the level of silencing is not high. Similarly siRNAs could be produced from *hpGus* RNA transcribed from an endogenous promoter (as in line S5) or a promoter of an actively expressed resident transgene (as in line S34). Furthermore, siRNAs in line S5 were capable of inducing effective silencing of the super-transformed *Gus* gene. Thus, *F. oxysporum* contains RNA silencing machineries required for both dsRNA processing and for siRNA-directed silencing, which is consistent with the identification of multiple Dicer and Argonaute-like genes from the *Fusarium oxysporum* strain 4287 genome using bioinformatics [see Additional file 1: Table S2].

A question is therefore why hpRNA transgenes investigated here were not effective at generating siRNAs and inducing silencing in *F. oxysporum*. Three independent studies in *S. pombe* utilizing the *Ura4* gene as a target have shown that hpRNA transgenes can direct both heterochromatin formation (transcriptional gene silencing) and post-transcriptional gene silencing, depending on the location of the targeted gene within the host genome [14,25,26]. This suggests that an hpRNA transcript in fission yeast can activate two independent gene silencing pathways, transcriptional and post-transcriptional. It is noteworthy that fission yeast contains only a single set of RNA silencing proteins which mediate both heterochromatin formation and post-transcriptional silencing, suggesting that these two pathways are mechanistically linked. It is possible that hpRNA also induces transcriptional silencing in *F. oxysporum*, which can target the hpRNA transgene itself to cause transcriptional self silencing. A recent study in plants has indicated that hpRNA transgenes are subject to self silencing through siRNA-directed DNA methylation, a plant-specific transcriptional gene silencing pathway [37]. Northern blot analysis indicated that the *hpGus* and *hpFrp* transgenes were poorly transcribed in the transgenic *F. oxysporum* lines, suggesting that they were transcriptionally silenced. However, a DNA methylation analysis of the *hpGus* transgene failed to detect any methylation at either locus (data not shown), suggesting that DNA methylation is not involved in the transcriptional silencing, but that histone modification might be responsible as in the case of transcriptional silencing in fission yeast [14].

While the strong and constitutive *gpdA* promoter of the hpRNA constructs failed to confer high levels of hpRNA and siRNA expression in *F. oxysporum*, the *hpGus* RNA was efficiently transcribed by an endogenous element, presumably the promoter of an actively expressed endogenous gene, which was accidentally acquired by T-DNA integration. Furthermore, the *gpdA* promoter of the resident *Gus* target gene was also able to transcribe the hpRNA formed by DNA rearrangement between the *Gus* and the *hpGus* transgenes. This implies that promoters of newly introduced hpRNA transgenes are more susceptible to transcriptional silencing than those of genes already residing in the genome which are actively expressed. Consistent with this possibility, transgene promoters are highly susceptible to hpRNA-induced transcriptional inactivation in plants whereas endogenous promoters are usually resistant to hpRNA-induced transcriptional silencing [11].

Our results suggest that non-polyadenylated dsRNA is efficiently processed by Dicer into siRNAs in *F. oxysporum*. Lines S5 and S34 both produced small RNA species and both lacked transcription terminators for hpRNA transcription and are therefore likely to produce non-polyadenylated precursor transcripts. Furthermore, bi-directional transcription of a *Gus* sequence from convergent promoters (*conP-Gus*) without transcription terminators consistently downregulated *Gus* mRNA levels. It is possible that Dicer processing of dsRNA occurs in the nucleus of *F. oxysporum*, and therefore non-polyadenylated dsRNA is a preferred substrate because of its possible retention in the nucleus after transcription. However, further work is needed to test this idea. Also, as no siRNAs were detectable in plants transformed with the convergent promoter construct, it cannot be ruled out that an alternative, siRNA-independent, mechanism may account for the *conP-Gus*-induced gene silencing.

## Conclusions

We demonstrate here that RNA silencing machineries exist in *F. oxysporum*, however conventional hpRNA transgenes are not effective at inducing gene silencing due to poor transcriptional activity of the transgene. Convergent promoter transgenes are capable of inducing gene silencing, but with low silencing efficiency. Future studies should focus on achieving potent and consistent RNA silencing in *F. oxysporum* by preventing transcriptional silencing of hpRNA or other types of dsRNA transgenes. Alternatively, the transcriptional silencing mechanism may be exploited to develop effective gene silencing technology in *F. oxysporum*.

## Methods

### Media and solutions

All chemicals and media were obtained from either Sigma (Sydney, NSW, Australia) or BDH (VWR International, Radnor, PA, USA). Hybond membranes were obtained from Amersham Biosciences (GE Healthcare Australia, Rydalmere, NSW, Australia). Potato Dextrose Agar (PDA, Sigma) and Potato Dextrose Broth (PDB, Sigma) were both used at half strength and PDA was supplemented with 12.5 g/l agar. PDA or PDB containing 0.1 M Tris- HCl pH 8 was used for phleomycin selection. Luria Bertani (LB) medium contained per liter 5 g yeast extract, 5 g tryptone and 10 g NaCl, supplemented with 15 g/l agar for solid media. Induction Medium contained 10 mM KH_2_PO_4_, 10 mM K_2_HPO_4_, 2.5 mM NaCl, 4 mM (NH_4_)_2_SO_4_, 0.5% glycerol, 9 μM FeSO_4_, 10 mM glucose, 40 mM MES buffer pH 5.3, 0.7 mM CaCl_2_ and 2 mM MgSO_4_. Induction agar was the same as induction medium except it contained 5 mM glucose and 0.2 μM acetosyringone. SDS/BSA hybridization solution contained per liter 70 g SDS, 10 g BSA, 122.4 g Na_2_HPO_4_ × 12 H_2_O, 25 g NaH_2_PO_4_ and 10 mM EDTA pH 8.0. Northern hybridization solution contained 50% formamide, 1% SDS, 5 × SSPE buffer (3 M NaCl, 0.2 M NaH_2_PO_4_ and 0.02 M EDTA pH 7.4) and 5 x Denhardt’s solution (2% Ficoll 400, 2% PVP and 2% BSA). DNA extraction buffer contained 0.5 M NaCl, 0.1 M Tris- HCl pH 8.0 and 50 mM EDTA pH 8.0. protein extraction buffer contained 50 mM NaPO_4_, 10 mM EDTA pH 7.0, 0.1% Triton X-100, 0.1% Sarkosyl and 10 mM ß-mercaptoethanol. MUG assay buffer was protein extraction buffer containing 2 mM 4-methylumbelliferyl-ß-D-glucoronide hydrate (MUG).

### Growth and storage of *Fusarium* and bacteria

*Fusarium oxysporum* f.sp. *conglutinans* strain 5176 was grown at 28°C in either liquid PDB shaking at 200 rpm or on solid PDA. For long-term storage of *Fusarium*, conidia of 1 ml from a liquid culture were collected by centrifugation, suspended in 500 μl of 15% glycerol and stored at −80°C.

*Agrobacterium tumefaciens* strain AGL0 was used for *Fusarium* transformation as it produced the largest number of transformants compared to other strains. AGL0 was grown at 28°C in liquid LB medium or on solid LB plates supplemented with 20 μg/ml rifampicin and the appropriate antibiotic to select for the binary vector. *E. coli* strain DH5α was used for construction, propagation and amplification of plasmid DNA and was grown in liquid or on solid LB medium supplemented with the appropriate antibiotic at 37°C.

### Creation of the β-glucuronidase (Gus) expression cassette

For all primer sequences see Additional file 1: Table S1. The *gpd*A promoter sequence [Genbank Z32524] was amplified using the *gpd*A-F1 primer containing a terminal *Eco*RI site and the *gpd*A-R1 reverse primer carrying the restriction enzyme recognition sites *Afe*I, *Afl*II, *Eco*RV, *Xba*I, *Hin*dIII and *Kas*I. Similarly, the *trp*C terminator sequence [Genbank X02390] was amplified using the *trp*C-R1 reverse primer containing a terminal *Kpn*I site and the *trp*C-F1 forward primer carrying the enzyme recognition sites *Xba*I, *Hin*dIII, *Kas*I, *Hpa*I, *Cla*I and *Bam*HI. The two fragments were combined into a single construct by overlapping PCR, thus creating a multiple cloning site (MCS) located between the *gpd*A promoter and *trp*C terminator. The fusion fragment was ligated into pGEM-T Easy (Promega, Madison, WI, USA), verified by sequencing and termed pUS1.

The coding sequence of the bacterial *UidA* (*Gus*; [Genbank AAC74689]) gene was ligated into the *Eco*RV site of plasmid pUS1 and the entire cassette excised using *Eco*RI. The overhangs were end-filled with *Pfu* (Promega) polymerase and the fragment ligated into the *Eco*RV site of the fungal binary vector pPZPHyg [38]. The resulting vector was verified by sequencing and termed pPZPHyg-*Gus* (Figure 1).

### Creation of hairpin RNA silencing constructs

The fungal binary vector pKR1 was based on vector pRW1p [39], which was extended to contain a MCS flanked by the *gpd*A promoter and *trp*C terminator. Using *Pfu* polymerase and the primer pair *trp*C-F2 and *trp*C-R2, the *trp*C terminator was amplified by PCR and ligated into the *Eco*RV site of vector pBC sk + (Stratagene), creating pBC-*trp*C. A *Spe*I recognition site was introduced into pAN9-1 (a derivative of vector pAN7-1 [40]) immediately 3′ of the *gpd*A promoter by site-directed mutagenesis. The *gpd*A promoter was then excised using *Eco*R1 and *Spe*I, end-filled using *Pfu* polymerase and ligated into the *Sma*I site of pBC-*trp*C to create pBC-*gpd*A:*trp*C. The *gpd*A:*trp*C fragment was then removed from pBC-*gpd*A:*trp*C using *Xba*I and *Hin*dIII and end-filled. The vector pRW1p was restricted with *Eco*RI and *Bam*HI, end-filled and re-ligated to remove several endonuclease recognition sites. The vector was then digested with *Xba*I and *Hin*dIII, end-filled and ligated with the *gpd*A:*trp*C fragment from plasmid pBC-*gpd*A:*trp*C to create the vector pRW1p-*gpd*A:*trp*C. The *lac*Z gene was PCR amplified with *Pfu* polymerase using the primers *lac*Z-F and *lac*Z-R, which carried the recognition sites of 14 unique restriction enzymes, thus creating a *lac*Z gene flanked by a MCS. This fragment was ligated into the *Sma*I site of vector PSP72 (Promega), released from PSP72 by digestion with *Eco*RI and *Mun*I and then ligated into the *Eco*RI site of pRW1p-*gpd*A:*trp*C. The resulting vector was then restricted with *Hin*dIII and re-ligated to excise the *lac*Z gene, leaving the MCS in place and creating the binary vector pKR1.

The construction of the *hpGus* sequence was described previously [27]. Basically, the *Gus* gene, which contained two *Eco*RV sites at 562 nt and 793 nt, was digested with *Eco*RV and re-ligated to remove the internal 231 nt *Eco*RV region. This was to prevent the expression of a functional Gus protein. This 231 bp region is therefore unique to the *Gus* gene and not present in the *hpGus* gene and was used as a template for the preparation of radioactive probes to distinguish between the *hpGus* and full length *Gus* transcripts. The resulting fragment (approximately 1.6 kb) was ligated at the 3′ end to a 606 bp 5′ *Gus* fragment (up to the first HincII site of the *Gus* ORF) in an antisense orientation, forming an inverted repeat (or *hpGus* sequence) containing a approximately 560 bp complementary sequence interrupted by a 1.1 bp *Gus* fragment (Figure 1). The *hpGus* gene was transferred from the pGEM-T Easy vector into pKR1 using *Eco*RI and *Apa*I.

To create the *hpGfp* construct for expression in fungi, an existing *hpGFP* sequence was excised from vector pUQC218 [41]*Eco*RI digestion, end-filled with *Pfu* DNA polymerase, and ligated into the *Eco*RV site of pUS1. The resulting expression cassette was then excised using *Eco*RI and ligated into the *Eco*RI site of pRW1p to create the binary vector pRW1p-*hpGfp*. This vector mediates expression of an hpRNA that contains the *pdk* intron in spliceable orientation (Figure 1).

The *hpFrp* gene was constructed in similar fashion to the *hpGus* gene. A long *Frp* fragment (nt 39–1063 of the *Frp*1 gene [Genbank AY673970]) was amplified by PCR using the forward primer *frp*L-F with a 5´ terminal *Afl*II recognition site and the reverse primer *frp*L-R with a 5´ terminal *Hin*dIII recognition site. A short *frp* fragment was PCR amplified using the forward primer *frp*S-F with a 5´ terminal *Bam*HI site and the reverse primer *frp*S-R with a 5´ terminal *Hin*dIII site. The two fragments were successively ligated into the *Afl*II/*Hin*dIII and *Hin*dIII/*Bam*HI sites of pUS1. The cassette was released via *Eco*RI digestion, the overhangs filled using *Pfu* polymerase and the fragment ligated into the *Eco*RV site of vector pPZPhyg to create pPZPhyg-*hpFrp* (Figure 1). The 3′ region (nt 1064 onward) of the *frp*1 gene was not included in *hpFrp* and the terminal 492 nt (nt 1090 onward) were used as a template for synthesis of radioactive probes to differentiate between the *hpFrp* gene and endogenous *Frp1* transcript.

### Creation of convergent promoter silencing construct

The *trp*C promoter and *trp*C terminator were amplified by PCR using primers *trp*C-PrF, *trp*C-PrR and *trp*C-TF, *trp*C-TR, respectively. The two fragments were combined by overlapping PCR, creating a promoter:terminator sequence (pro:ter) interrupted by *Apa*I and *Eco*RI restriction sites. After cloning into the pGEM-T Easy vector, the pro:ter fragment was transferred into the pPZPnat1 vector [GenBank:AY631958] using *Xba*I and *Pst*I to create pPZPnat-pro:ter. Then, the 1.1 kb 3′ region of the *Gus* gene was excised from a pGEM-T Easy vector carrying the *Gus* ORF and ligated into vector pPZPnat-pro:ter via the *Apa*I and *Eco*RI sites. Subsequently, the *gpd*A promoter was excised from pUS1 using *Bam*HI and *Pst*I and ligated behind the *trp*C terminator sequence. This was achieved such that the *gpd*A promoter and the *trp*C promoter were in convergent orientation (Figure 8). To create the final *conP-Gus* construct, the *trpC* terminator sequence was deleted through *Eco*RI and *Bam*HI digestion, and the remainder of the plasmid was end-filled using *Pfu* polymerase and re-ligated (Figure 8). All plasmids were verified by sequencing prior to use.

### *Fusarium* transformation

Transformation of *Fusarium* was achieved by co-cultivation of conidia with *Agrobacterium* adapted from [42]. AGL0 carrying the binary vector of interest was grown in 7.5 ml of LB medium with appropriate antibiotics for two days at 28°C, the cells of 1 ml of the culture collected by centrifugation and suspended in 20 ml of induction media. Cells were incubated at 28°C for a further 6 h. *Fusarium* conidiospores were grown in PDB for two days, filtered through miracloth (Calbiochem, Merck KGaA, Darmstadt, Germany) and the optical density at 600 nm (OD_600_) measured. The spore content was calculated using a standard curve. Conidia were collected by centrifugation at 4,000 rpm for 10 min, suspended in water and the concentration adjusted to 1E^6^ spores/ml. Then, 100 μl *Fusarium* spores were mixed with 400 μl AGL0 and 300 μl plated on induction agar overlayed with a Hybond membrane. After 48 h incubation at 28°C the membranes were transferred onto PDA containing either 50 μg/ml hygromycin or 50 μg/ml nourseothricin as well as 100 μg/ml timentin, or 25 μg/ml phleomycin and 250 μg/ml of cefotaxime. Cefotaxime selection was substituted with timentin after the first round of selection. Individual transformants were transferred onto fresh selective medium. Genetically pure cultures were obtained by plating 25 μl of a spore suspension (1E^3^ spores/ml) on a selective plate and subsequent isolation of a star colony.

### DNA and RNA isolations

To generate the biomass required *Fusarium* mycelium was obtained and harvested as described in [43]. For DNA isolations mycelium was ground in liquid N_2_ and the powder suspended in 500 μl DNA extraction buffer and 50 μl of 10% SDS and incubated at 50°C for 10 min. The DNA was extracted with 500 μl phenol/chloroform and subsequently with 500 μl chloroform and ethanol precipitated at −20°C overnight. The pellet was washed with 75% ethanol, air-dried and the DNA suspended in TE buffer containing RNase.

RNA was isolated using Trizol Reagent according to manufacturer’s instructions and the pellets suspended in either water or formamide. DNA and RNA concentrations were measured using the NanoDrop ND-1000 (Thermo Fisher Scientific, Waltham, MA, USA).

### Southern blot analysis

A total of 5 μg of genomic DNA was digested overnight, the DNA phenol/chloroform purified and ethanol precipitated. The fragments were separated on a 1% agarose gel at 2 V/cm over night. The gel was incubated in 0.25 M HCl for 10 min, then in 1.5 M NaCl and 0.5 M NaOH for 30 min, followed by a 30 min washing step in 1.5 M NaCl and 0.5 M Tris- HCl pH 7.5. The DNA fragments were transferred to Hybond-N^+^ membrane by standard capillary transfer in 20 × SSC over night and then cross-linked to the membrane using a UV crosslinker (Stratagene, Agilent Technologies, Mulgrave, VIC, Australia). Pre-hybridization was carried out in SDS/BSA hybridization solution for at least 3 h prior to adding the probe.

Probes incorporating ^32^P-dCTP were prepared using the Megaprime DNA Labelling System (Amersham) according to manufacturer’s instructions. The probes were purified using Amersham G50 columns, denatured at 100°C for 5 min, kept on ice for 10 min and then added to the membrane. Hybridization was carried out in approximately 20 ml SDS/BSA Hybridization solution at 65°C over night. Membranes were washed three times in 2 × SSC + 0.1% SDS for 10 min at 65°C before exposure to a Phosphorscreen.

### Northern blot analysis

For mRNA northern blotting 10 μg of total RNA was separated on a 1.3% agarose formaldehyde gel and the fragments transferred to Hybond-N membranes by standard 20 × SSC capillary transfer over night. The RNA was cross-linked to the membrane in a UV crosslinker and the membrane pre-hybridized at 42°C in northern hybridization buffer for at least 3 hours. Hybridization was carried out at 42°C overnight and membranes were washed twice in PES buffer (0.04 M NaPO_4_, 0.2% SDS and 1 mM EDTA pH 7.2) at 65°C before exposure to a Phosphorscreen. For siRNA northern analysis, 15 μg of total RNA were separated on a 17% polyacrylamide-urea gel and the fragments transferred to Hybond-N^+^ membrane by electro-blotting. The RNA was cross-linked to the membrane by UV crosslinking. All hybridization and washes were carried out at 42°C. Membranes were pre-hybridized in northern hybridization buffer for at least 3 hours prior to adding the probe. Hybridization was carried out overnight and membranes washed twice in 2 × SSC + 0.2% SDS prior to exposure to a Phosphorscreen.

Probes were prepared by *in vitro* transcription incorporating ^32^P-UTP using the Riboprobe Combination System (Promega) according to manufacturer’s instructions. Probes were precipitated with 7.5 M ammonium acetate and suspended in 20 μl TE buffer. Probes for siRNA northern analysis were treated with a carbonate solution (80 mM NaHCO_3_ and 120 mM Na_2_HCO_3_) at 60°C and then precipitated with 7.5 M ammonium acetate. Screens were developed using a Phorphorimager (FLA-5000; Fujifilm Corporation, Tokyo, Japan).

### Gus staining and MUG assay

Gus activity in mycelial fractions was assessed by incubating samples with X-glucuronide solution (0.1 M NaPO_4_, 10 mM EDTA pH 7.0, 0.5 mM potassium ferricyanide, 0.5 mM potassium ferrocyanide, 1 mM X-Glucuronide and 0.1% Triton X-100) at 37°C for several hours or overnight.

For quantitative Gus activity analysis, a small sample of mycelium was obtained as described in [43] and ground with sand in 50 to 100 μl protein extraction buffer for 30 s using a glass rod and drill. The samples were centrifuged at 4°C for 5 min and the protein concentration of the supernatant measured by standard Bradford Assay. MUG assay was performed as described previously [44] and the Gus activity determined from the slope of the curve using Excel. Gus activity in each sample was calculated relative to the total amount of protein in the cell extracts.

### Cleavage product identification by rapid amplification of cDNA ends (5´ RACE)

A DNA/RNA adapter was ligated to the free 5′ phosphates of DNase treated total RNA samples. Four μg of total RNA were incubated with 40 pmol of adapter, 50 mM HEPES buffer pH 7.5, 1 mg/ml BSA, 8% glycerol, RNaseOut (Invitrogen, Life Technologies Australia, Mulgrave, VIC, Australia) and T4 RNA ligase (Promega) in 1 × T4 RNA ligase buffer for 2 h at room temperature (RT). The RNA was phenol/chloroform extracted and suspended in 12 μl RNase-free water. Reverse transcription of 6 μl of ligate was carried out using gene specific primers *Gus*-RT1 or *Gus*-RT2. RACE products were amplified by PCR using an adapter primer and a gene-specific nested primer (*Gus*-RT1n or *Gus*-RT2n) and obtained fragments were separated by agarose gel electrophoresis. Fragments were excised from the gel, eluted using the Ultra Clean DNA Purification Kit (Mo Bio Laboratories, Carlsbad, CA, USA) and ligated into the pGEM-T Easy vector for sequencing.

### Reverse transcription

Total RNA samples were treated with RNase-free DNase One to remove all contaminating genomic DNA. Samples were analyzed for purity by PCR of an endogenous gene prior to reverse transcription. Reverse transcription was carried out using gene-specific primers and SuperScript III Reverse Transcriptase (Invitrogen) according to manufacturer’s instructions. For subsequent PCR reactions 0.5 μl of cDNA was used as template.

### Creation of a lambda phage library

The library was prepared from genomic DNA of *Fusarium oxysporum* line S34. The library was created using the Lambda Dash II/*Bam*HI vector kit (Invitrogen). All steps were performed according to manufacturer’s instructions. Genomic DNA was partially digested using *Sau*3A and size fractionated by centrifugation through a sucrose gradient. The layer containing fragments of approximately 10 kb size was used for ligated into the pre-digested lambda vector to create the library. Plaque lifts and subsequent DNA blotting was performed to identify phage plaques that carry *Gus*-specific sequences. Phage DNA from an individual pure phage lysate was extracted as described in [45]. Entire lambda phage was sequenced to determine the nature of the *Gus*-specific region.

### Sequencing

Plasmid DNA was sequenced using Big Dye Terminator v3.1 (Applied Biosystems, Life Technologies Australia, Mulgrave, VIC, Australia) according to manufacturer’s instructions. Reactions were ethanol precipitated, run using a 96 capillary 3730 DNA Analyser (Applied Biosystems) at the John Curtin School of Medical Research, Australian National University, Canberra and analyzed using the Vector NTI program suite. Sequencing of the lambda phage DNA was carried out using the Ion Torrent Platform at the John Curtin Institute, Australian National University, Canberra and analyzed using the CLC Genomics Workbench (CLC bio, Taipei, Taiwan).

## Abbreviations

Dcl: Dicer-like protein; disiRNAs: Dicer-independent small interfering RNAs; dsRNA: double-stranded RNA; Frp1: *Fusarium oxysporum* F-box protein required for pathogenesis 1 gene; Gfp: green fluorescent protein gene; gpdA: promoter of the *Aspergillus nidulans* glyceraldehyde 3-phosphate dehydrogenase gene; Gus: *Escherichia coli* β-glucuronidase reporter gene; hpGus: β-glucuronidase hairpin RNA transgene (*hpGus*); hpRNA: hairpin RNA; kb: kilo base pair; MCS: multiple cloning sites; miRNAs: micro RNAs; milRNA: miRNA-like genes; MUG: 4-methylumbelliferyl-β-D-glucuronide; qiRNAs: Qde2-interacting siRNAs; RISC: RNA-induced silencing complex; siRNA: small interfering RNA; trpC: transcription termination sequence or promoter sequence of the *Aspergillus nidulans* tryptophan synthase gene.

## Competing interests

The authors declare that they have no competing interests.

## Authors’ contributions

US helped design the study, carried out molecular studies and bioinformatics analyses and drafted the manuscript. NAS carried out molecular work, had intellectual input into the study design and helped draft the manuscript. KK and MA critically revised the manuscript. MA helped create the lambda library. MBW designed this study and drafted and revised the manuscript. All authors read and approved the final manuscript.

## Supplementary Material

Additional file 1 Supportive information. Click here for file
