# Supplementary material for: Analysis of hairpin RNA transgene-induced gene silencing in Fusarium oxysporum
Source: Silence. 2013 Jul 2;4:3. doi: 10.1186/1758-907X-4-3 (PMC3733888; doi:10.1186/1758-907X-4-3)
Supplement: Additional file 1 — Supportive information. [file 1758-907X-4-3-S1.zip › 2037588831959193_add4.pdf]

Table S1: Primers used in this study. Italic characters represent RNA sequence.

Name	5′ to 3′ sequence
*gpd*A-F1	gAATTCCCTTgTATCTCTACACACAggC
*gpd*A-R1	AACggCgCCAAgCTTCTAgATATCTTAAgCgCTCTgCTCAAgCgg ggTAgC
*trp*C-F1	TCTAgAAgCTTggCgCCgTTAACATCgATCgggATCCACTTAACg
*trp*C-R1	ggTACCCgATTTCgACgAgCCCTC
*trp*C-F2	ACTTAACgTTACTgA
*trp*C-R2	CgACTAgTCgATTTCgTCgACCCTCTAAAC
*lac*Z-F	gAATTCTAgATATCgggCCCgCggCgCgCCTACgTAAgCTTAACTg ACAgAACCgCAAC
*lac*Z-R	CAATTggTACCgTTAACTAgTAgATCTTAATTAAgCTTgACgTAT gTgCTTAgCTC
*frp*L-F	CTTAAggCCACATATCACCgAATTTgC
*frp*L-R	AAgCTTAAAgCCTTTCCACTTgTCgC
*frp*S-F	ggATCCgCCACATATCACCgAATTTgC
*frp*S-R	AAgCTTCgTTgAgCTTCAgTTTggCg
*trp*C-PrF	ACCACTCTAgAgACCAgAAgATgACATTgAAgg
*trp*C-PrR	TTCAgTAACgTTAAgTggAATTCAgggCCCATgCTTgggTAgAATA gg
*trp*C-TF	CCTATTCTACCCAAgCATgggCCCTgAATTCACTTAACgTTACT

	gAA
*trp*C-TR	AAgCTTCTgCAgAATggATCCATTTCgACgAgCCCTCTAAAC
DNA/RNA adapter	AATTAACCCTCACTAAAggCATCgT*AggCACCUgAAA*
Gus-RT1	gTgCCTTgTCCAgTTgCAACC
Gus-RT1n	ACCTgTTgATCCgCATCACgC
Gus-RT2	CCCCAATCCAgTCCATTAATgCg
Gus-RT2n	gTgCACCATCAgCACgTTATCg
Gus-RT3	gTCgTCggTgAACAggTATgg
A-RT2	CTTAAgTTCgCCCTTCCTCCC
A-RT3	TAACAgCTACCCCgCTTgAgC

Table S2: Identification of RNA silencing machinery genes in the *Fusarium oxysporum* genome. Known fungal RNA silencing machinery proteins were used for BLASTP search of the published *Fusarium oxysporum* genome sequence (Broad Institute). Shown here are the identified *Fusarium oxysporum* RNA silencing machinery gene as well as genes from selected fungal species. It has been shown that the fungal RNA silencing machinery proteins cluster into two distinct clades, associated either with quelling or meiotic silencing, however some RdRP-like proteins do not fall into either cluster [47,16]. Clustering of newly identified *Fusarium oxysporum* proteins was determined by alignment of the here listed proteins using the E-INS-i algorithm of MAFFT [48] and a phylogenetic tree (neighbor joining, 500 bootstraps) was constructed using MEGA5 [49]. Proteins are listed here according to their clustering with *N. crassa* proteins.

**Dicer-like genes**	**Quelling**	**Meiotic silencing**	**Not clustering**

Fusarium oxysporum	FOXG_13826.2	FOXG_09093.2	
Magnaporthe oryzae	dcl-2 (MGG_12357)	dcl-1 (MGG_01541)	
Neurospora crassa	dcl-2 (NCU06766)	sms-3 (NCU08270)	
Aspergillus fumigatus	dcl-2 (AFUA_4G02930)	dcl-1 (AFUA_5G11790)	
Schizosaccharomyces pombe		dcr1 (SPCC188.13c)	
**Argonaute-like genes**			
Fusarium oxysporum	FOXG_03010.2	FOXG_00711.2	
	FOXG_16455.2		
	FOXG_14081.2		
	FOXG_12456.2		
Magnaporthe oryzae	MGG_01294	MGG_14873	
	MGG_13617		
Neurospora crassa	qde-2 (NCU04730)	sms-2 (NCU09434)	
Aspergillus fumigatus	qde-2 (AFUB_082250)		
Schizosaccharomyces pombe		ago1 (SPCC736.11)	
**RdRP-like genes**			
Fusarium oxysporum	FOXG_02461.2	FOXG_03081.2	FOXG_11123.2
			FOXG_16453.2
Magnaporthe oryzae	MGG_13453	rdrp-1 (MGG_02748)	MGG_06205
Neurospora crassa	qde-1 (NCU07534)	sad-1 (NCU02178)	rrp-3 (NCU08435)
Aspergillus fumigatus		Sad-1 (AFUA_3G06790)	AFUA_5G09430
Schizosaccharomyces pombe		rdp1 (SPAC6F12.09)	
