# Supplementary figures and images for: Analysis of hairpin RNA transgene-induced gene silencing in Fusarium oxysporum
Source: Silence. 2013 Jul 2;4:3. doi: 10.1186/1758-907X-4-3 (PMC3733888; doi:10.1186/1758-907X-4-3)

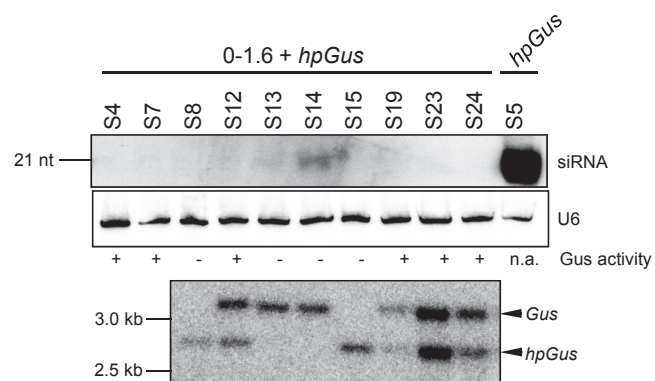

Supplement: Additional file 1 — Supportive information. [file 1758-907X-4-3-S1.zip › Schumann et al_FigS1_revised2.pdf]

A

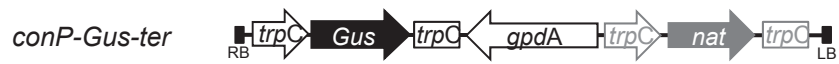

B

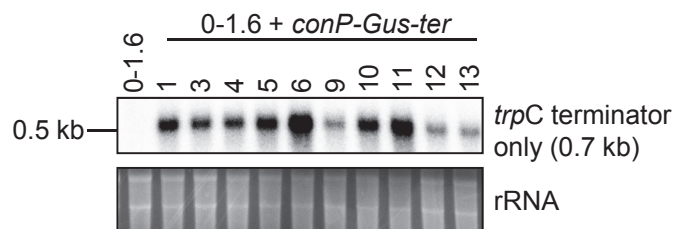

C

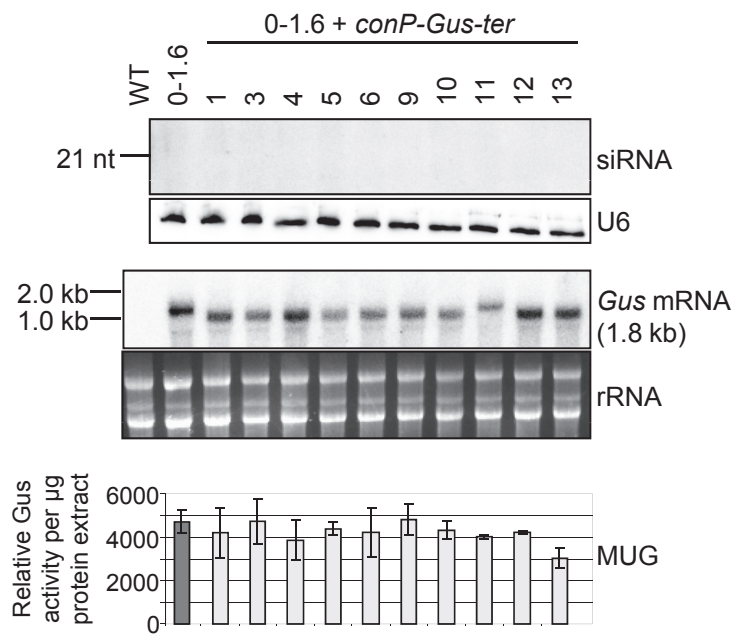

Supplement: Additional file 1 — Supportive information. [file 1758-907X-4-3-S1.zip › Schumann et al_FigS2_revised2.pdf]

A

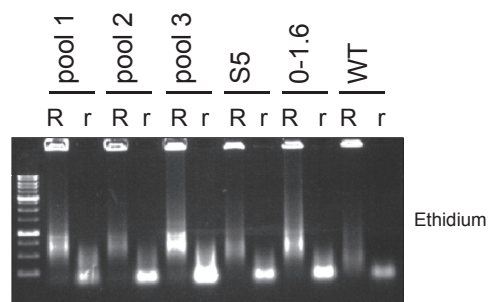

B

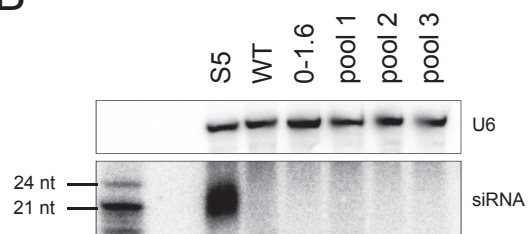

Supplement: Additional file 1 — Supportive information. [file 1758-907X-4-3-S1.zip › Schumann et al_FigS3_revised2.pdf]
